# Use of Cannabinoids to Treat Acute Respiratory Distress Syndrome and Cytokine Storm Associated with Coronavirus Disease-2019

**DOI:** 10.3389/fphar.2020.589438

**Published:** 2020-11-06

**Authors:** Prakash Nagarkatti, Kathryn Miranda, Mitzi Nagarkatti

**Affiliations:** ^1^ Department of Pathology, Microbiology and Immunology, University of South Carolina School of Medicine, Columbia, SC, United States; ^2^ University of South Carolina, Columbia, SC, United States

**Keywords:** severe acute respiratory syndrome coronavirus 2, coronavirus disease 2019, cannabinoid, acute respiratory distress syndrome, cytokine storm

## Abstract

Coronavirus disease 2019 (COVID-19) is a highly infectious respiratory disease caused by the severe acute respiratory syndrome coronavirus 2. A significant proportion of COVID-19 patients develop Acute Respiratory Distress Syndrome (ARDS) resulting from hyperactivation of the immune system and cytokine storm, which leads to respiratory and multi-organ failure, and death. Currently, there are no effective treatments against hyperimmune syndrome and ARDS. We propose that because immune cells express cannabinoid receptors and their agonists are known to exhibit potent anti-inflammatory activity, targeting cannabinoid receptors, and endocannabinoids deserve intense investigation as a novel approach to treat systemic inflammation, cytokine storm, and ARDS in patients with COVID-19.

## Introduction

Severe acute respiratory syndrome coronavirus 2 (SARS-CoV-2) has caused a pandemic triggering human misery, death, and severe economic loss. SARS-CoV-2 uses viral “spike” (S) protein to attach to a glycoprotein receptor called the angiotensin-converting enzyme 2 (ACE2) which is widely expressed in the body not only on cells in the lower respiratory tract but also in the cardiovascular system, kidneys, central nervous system, adipose tissue, and the gastrointestinal tract (Gheblawi et al., 2020; Shang et al., 2020; Wu et al., 2020a). This receptor facilitates the infection by SARS-CoV-2 in the respiratory tract and spread to other tissues. While the precise nature of innate and adaptive immune response triggered by SARS-CoV-2 remains to be elucidated, initial studies have shown that the host produces neutralizing antibodies that can bind to the virus and prevent it from attaching to ACE2, thereby inhibiting the virus from infecting additional host cells (Wu et al., 2020b). Additionally, coronavirus disease 2019 (COVID-19) patients also produce CD4^+^ Th responses that help B cells to produce antibodies as well as CD8^+^ cytotoxic T cells that kill SARS-CoV-2-infected cells (Grifoni et al., 2020). The fact that the initial immune response is effective against SARS-CoV-2 is suggested by the fact that a majority of people infected with the virus recover completely from the infection.

The effectiveness of the immune system in clearing the infection is also evident from the fact that, in individuals who are immunocompromized such as in elderly people or people with chronic inflammation including those with metabolic syndrome, the inability to generate an effective antiviral immune response can lead to increased viral replication and spread across the respiratory and other systems. This can cause a more severe form of the disease leading to hyperactivation of the immune system and acute respiratory distress syndrome (ARDS) (Mehta et al., 2020; Pedersen and Ho, 2020).

## What Triggers Cytokine Storm and Acute Respiratory Distress Syndrome in Coronavirus Disease 2019 Patients?

The reason why majority of the population overcomes COVID-19 with mild or moderate symptoms and a small proportion develop ARDS leading to hospitalization is unclear. ARDS is defined as a form of respiratory failure that is caused by a variety of insults such as pneumonia, sepsis, trauma and certain viral infections. The common feature of ARDS includes systemic hyperactivation of immune response leading to inflammation in the lungs followed by the development of pulmonary edema, alveolar damage, and respiratory failure (Fan et al., 2018). There are over 200,000 people affected by ARDS annually in the US and three million people globally, and ARDS causes over 75,000 deaths in the US alone (37.5% mortality) (Fan et al., 2018). This number is certainly going to increase significantly with the COVID-19 pandemic. Currently, there are no pharmacological agents approved by FDA to treat ARDS because of which there is a high rate of mortality.

ARDS can result from a wide range of insults, and the precise nature of antigens or factors that trigger hyperactivation of the immune response, is unclear. The most challenging question which remains unanswered is whether hyperactivation of immune response seen in patients with the severe form of COVID-19 results from hyperimmune response against the virus or against secondary infections seen in these patients or a combination of both. It is less likely that the hyperactivation of the immune response is against the virus itself because these are the same patients who are immunocompromized that fail to exhibit an optimum response to the virus. However, it can be reasoned that an initial failure to mount an optimum response leads to rapid spread of the virus throughout the body which triggers hyperimmune response even in immunocompromized individuals. There is also evidence to support the second possibility that hyperactivation on immune response may result from secondary infections. The prevalence of coinfection varies among COVID-19 patients as indicated in different studies, but it can account for up to 50% among patients who die from COVID-19 (Lai et al., 2020). The copathogens include, *Streptococcus pneumoniae*, *Staphylococcus aureus*, *Klebsiella pneumoniae*, and the like (Lai et al., 2020). Some of these bacteria produce toxins such as Staphylococcus enterotoxin B (SEB) which can activate a large proportion of T cells, thereby causing cytokine storm, ARDS, and multiorgan failure (Lang et al., 2003). Also, as the coronavirus virus spreads, it is likely that the infection and the inflammation that ensues disrupt the normal microbiota found in the lungs, which leads to dysbiosis and emergence of pathogenic organisms, causing pneumonia and sepsis. Such dysbiosis has been shown during influenza and other respiratory viral infections (Hanada et al., 2018). The inflammatory cytokines can also activate innate immunity including macrophages which can cause significant damage to the lungs and other tissues (Lucas et al., 2020).

In an early study, it was observed that COVID-19 patients who develop the severe form and are hospitalized and exhibit several inflammatory cytokines including IL-2, IL-7, IL-10, G-CSF, IP-10, MCP1, MIP1α, and TNF (Huang et al., 2020). Also, IL-6 levels keep increasing and exhibit higher levels in patients who die versus those who survive (Zhou et al., 2020). Additionally, characterization of immune cells in the airways of COVID-19 patients showed that patients with severe or critical infection had greater abundance of monocyte-derived macrophages expressing high levels of inflammatory cytokines such as interleukin (IL)-8, IL-6, and IL-1b and chemokines, CCL2, CCL3, CCL4, and CCL7, which promoted additional recruitment of monocytes and neutrophils (Liao et al., 2020). Conversely, patients with moderate infection had airway macrophages expressing more T-cell-attracting chemokines such as *CXCL16* (Liao et al., 2020), with T cells playing a critical role to protect against SARS-CoV-2. Another mechanism of cytokine storm may involve active infection of immune cells such as the T cells, macrophages, and dendritic cells by SARS-CoV-2, which may result in aberrant cytokine production while also inducing death in such cells, thereby accounting for lymphopenia seen in COVID-19 patients (Gu et al., 2005). Thus, irrespective of the trigger and mechanisms involved, COVID-19 patients who develop ARDS exhibit hyperimmune state with cytokine storm (Mehta et al., 2020; Pedersen and Ho, 2020), which remains an important trigger leading to death (Tay et al., 2020).

## Current Therapies to Treat Cytokine Storm

Clinically, it is difficult to treat cytokine storm. Some of the therapeutic options to treat the hyperimmune state include steroids, intravenous immunoglobulin, selective cytokine blockade, specifically IL-6 (tocilizumab) or IL-1 receptor (anakinra), and JAK inhibition. However, thus far, none of these options are highly effective because the cytokine storm involves many inflammatory cytokines, and additionally, the anti-inflammatory treatments cannot overtly suppress immune response for the risk of increasing the primary or secondary infections in the patients (Mattos-Silva et al., 2020). While corticosteroids are not very effective against ARDS (Hough, 2014; Phua et al., 2020), recently, dexamethasone was shown to reduce overall mortality when compared with usual care (21.6% versus 24.6%) (Horby et al., 2020) and increase the number of ventilator-free days (WHO REACT Working Group et al., 2020). Thus, clearly, additional novel pharmacological approaches are in dire need to treat cytokine storm both in patients who develop ARDS due to COVID-19 or other etiologic agents.

## Cannabinoids as Anti-Inflammatory Agents?

Cannabinoids are a group of compounds that can activate two types of cannabinoid receptors designated CB1 and CB2. CB1 is highly expressed in the brain and other tissues, while CB2 is predominantly expressed in immune cells. However, studies have shown that CB1 is also expressed by cells of the immune system (Galiègue et al., 1995), and CB1 agonists can suppress inflammation (Do et al., 2004; Sido 2015a, Sido et al., 2015b). Immune cells as well as a wide variety of cells in the body produce cannabinoids called endocannabinoids which constitute a family of lipid transmitters derived from arachidonic acid that act as endogenous ligands for CB1 and CB2. The endocannabinoids are rapidly metabolized by enzymes such as fatty acid amide hydrolase (FAAH), involved in the hydrolysis for anandamide (AEA) and monoacyl glycerol lipase (MAGL) for 2-arachidonolyl glycerol (2-AG) (Cravatt et al., 1996; Dinh et al., 2002; Sido et al., 2015a; Shamran et al., 2017). The fact that the cells of the immune system express molecules of the endocannabinoid system suggests that cannabinoids play a critical role in the regulation of immune response (Sido et al., 2016b). *Cannabis* has over 100 phytocannabinoids including the well-characterized tetrahydrocannabinol (THC), which is psychoactive and can bind to and activate both CB1 and CB2 and cannabidiol (CBD) which is nonpsychoactive. Unlike THC, CBD does not activate CB_1_ and CB_2_ receptors because of which it does not exert psychotropic activity (Devinsky et al., 2014). However, recent studies have shown that CBD may act as a negative allosteric modulator at CB1 (Tham et al., 2019) and partial agonist at CB2 (Mechoulam et al., 2007). CBD is also known to act through PPARγ (O’Sullivan, 2016), 5-HT1A (Russo et al., 2005), TRPV1 (Campos et al., 2012), and GPR55 (Ryberg et al., 2007).

Studies from our laboratory over the past 2 decades as well as those from others have shown that cannabinoids including THC and cannabidiol (CBD) act as potent anti-inflammatory agents (Lombard et al., 2007; Hegde et al., 2008; Hegde et al., 2010; Jackson et al., 2014a; Rao et al., 2015; Sido et al., 2015c; Sido 2016a; Elliott et al., 2018; Al-Ghezi et al., 2019a; Al-Ghezi 2019b; Almogi-Hazan and Or, 2020; Mohammed et al., 2020a; Nichols and Kaplan, 2020). In fact, based on our studies (Hegde et al., 2008), CBD has been approved by FDA to treat autoimmune hepatitis as an orphan drug. THC, which has been more widely studied, has been shown to act through multiple pathways to attenuate inflammation including: 1) induction of Tregs and myeloid-derived suppressor cells (MDSCs) (Hegde et al., 2010; Jackson et al., 2014a; Sido et al., 2015c; Robinson et al., 2015; Elliott et al., 2018), 2) apoptosis in activated T cells and dendritic cells (Lombard et al., 2007; Rieder et al., 2010), 3) switch from Th1 to Th2 differentiation (Yang et al., 2014), 4) epigenetic alterations including alterations in the expression of miRNA and histone modifications in immune cells (Hegde et al., 2013; Yang et al., 2014; Sido et al., 2015c; Rao et al., 2015; Al-Ghezi et al., 2019b; Yang et al., 2019; Mohammed et al., 2020a), 5) reversal of dysbiosis triggered in the gut and lungs during inflammation (Al-Ghezi et al., 2019a), and 6) inhibition of cytokine storm induced by bacterial superantigens through regulation of suppressor of cytokine signaling 1 (SOCS1) and miRNA (Rao et al., 2015). Recently, we also observed that cannabinoids suppress macrophage-mediated inflammation by shifting myeloid differentiation toward anti-inflammatory MDSCs (Miranda et al., 2020). Together, these studies have suggested that cannabinoids act through multiple pathways and on a wide array of cells to suppress inflammation and thus may be ideally suited to suppress cytokine storm.

## Targeting Cannabinoid Receptors and Endocannabinoids to Dampen Cytokine Storm

Specifically, our studies have shown that THC is highly effective in preventing cytokine storm and ARDS in animal models (Rao et al., 2015; Mohammed et al., 2020a; Mohammed et al., 2020b; Mohammed et al., 2020c). *Staphylococcus aureus*, which produces SEB, a superantigen, is known to colonize the human respiratory tract and has been implicated in airway inflammation and acute lung injury. SEB acts as a superantigen by activating a large proportion of T cells expressing certain Vβ-specific T-cell receptors. Such an activation leads to cytokine storm, ARDS, and 100% mortality in mice (Rao et al., 2015; Mohammed et al, 2020b; Mohammed et al., 2020c). Studies from our lab have shown that treatment of mice with THC administered through intraperitoneal route leads to attenuation of inflammation in the lungs, cytokine storm, ARDS, and 100% survival (Rao et al., 2015; Mohammed et al., 2020a; Mohammed 2020b; Mohameed et al., 2020c). To investigate how quickly THC acts, we performed cytokine analysis 3 h, 6 h, and 24 h after SEB exposure and found that some cytokines peaked as early as 3 h, and we found that THC was able to suppress these cytokines as early as 3 h (Rao et al., 2015). We found that THC downregulates miRNA-17–92 cluster, specifically miRNA-18a, which targets PTEN, an inhibitor of the PI3K/Akt signaling pathway, thereby enhancing T-regulatory cells (Rao et al., 2015). More recently, we also noted that SEB causes similar dysbiosis in the lungs and the gut, while THC treatment reverses these effects (Mohammed et al., 2020b). Importantly, fecal transplants from THC-treated mice into naïve mice that lacked microbiota led to the protection of these mice from SEB-mediated ARDS (Mohammed et al., 2020b). The fact that THC can rescue mice from SEB-mediated mortality is highly significant and suggests that THC can be used to treat cytokine storm and ARDS in humans.

One of the drawbacks in the use of cannabinoids that activate CB1 receptors such as THC is their psychotropic property and classification as Schedule 1 drug, which makes it difficult to pursue clinical trials and prescription in a clinical setting. Nonetheless, THC has been approved by the FDA to treat nausea and promote appetite in cancer patients receiving chemotherapy and in HIV patients. Also, combination of THC and CBD has been approved as a drug for treating muscle spasticity multiple sclerosis in several parts of the world. Also, THC + CBD combination has been shown to suppress neuroinflammation in the experimental model of MS (Al-Ghezi et al., 2019b). However, because CB2 receptor activation does not trigger a psychoactive response and CB2 selective agonists have also been found to be immunosuppressive (Lombard et al., 2007; Singh et al., 2012; Leleu-Chavain et al., 2013; Tomar et al., 2015), such compounds may also be effective to suppress cytokine storm. In fact, in a recent study, we found that the effect of THC to attenuate cytokine storm was dependent, at least in part, due to activation of CB2 receptors (Mohammed et al., 2020a). It is also possible to increase the levels of endocannabinoids by blocking the enzymes that metabolize these compounds such as the use of fatty acid amide hydrolase (FAAH) inhibitors which enhances the levels of AEA. In fact, studies using FAAH inhibitor or mice deficient in FAAH have shown that such approaches lead to an increase in AEA and consequent suppression of autoimmune hepatitis triggered by a polyclonal activator of T cells which triggers cytokine storm (Hegde et al., 2008). Also, FAAH inhibitor can attenuate colitis through suppression of proinflammatory immune response through regulation of miRNAs expression (Shamran et al., 2017) or other pathways (Andrzejak et al., 2011; Sałaga et al., 2014). Lastly, administration of endocannabinoids such as AEA can also attenuate Th1 cell-mediated inflammation (Jackson et al., 2014b).

## Conclusion and Future Direction

The fact that cells of the immune system produce endocannabinoids and express both CB1 and CB2 cannabinoid receptors provides unique opportunities into investigating how the cannabinoid system can be engineered to suppress inflammation using both exogenous and endogenous cannabinoids. Because cannabinoids are potent suppressors of inflammation as evidenced by their ability to suppress cytokine storm in animal models, they may serve as novel therapeutic agents to treat cytokine storm and ARDS that are seen in patients with or without COVID-19. There is a dire need for novel anti-inflammatory agents that exert broad spectrum cytokine suppression associated with ARDS considering that currently up to 40% of such patients, including those with COVID-19, die because currently there are no FDA-approved drugs that are highly effective against cytokine storm and ARDS. While animal studies are striking, clinical trials with cannabinoids especially with THC are lacking because it is classified as Schedule 1 drug, which makes it difficult to pursue such studies. However, targeting CB2 receptors and modulation of endocannabinoids in patients may offer novel insights into their therapeutic efficacy against ARDS. COVID-19 is a complex disease, and while the nature and role of immune response against the virus is being actively studied, it is clear that hyperimmune response and cytokine storm seem to significantly contribute toward mortality. Thus, while all attempts are being focused on control of viral replication through development of vaccines, it is also critical to ensure that the vaccines do not trigger hyperactivation of immune response. It is equally important to develop new anti-inflammatory therapies that can effectively block the cytokine storm seen in patients with severe form of COVID-19. We believe that cannabinoids hold significant promise as potent anti-inflammatory agents.

## Author Contributions

Writing, review, & editing: PN, KM, and MN. Funding acquisition: PN and MN.

## Funding

The studies were supported in part by NIH grants P01AT003961, R01AT006888, R01AI123947, R01AI129788, R01MH094755, and P20GM103641 to MN and PN.

## Conflict of Interest

The authors declare that the research was conducted in the absence of any commercial or financial relationships that could be construed as a potential conflict of interest.
